# Genetic constraints predict evolutionary divergence in *Dalechampia* blossoms

**DOI:** 10.1098/rstb.2013.0255

**Published:** 2014-08-19

**Authors:** Geir H. Bolstad, Thomas F. Hansen, Christophe Pélabon, Mohsen Falahati-Anbaran, Rocío Pérez-Barrales, W. Scott Armbruster

**Affiliations:** 1 Centre for Biodiversity Dynamics, Norwegian University of Science and Technology, 7491 Trondheim, Norway; 2 Department of Biology, Norwegian University of Science and Technology, 7491 Trondheim, Norway; 3 Department of Biology, Centre for Ecological and Evolutionary Synthesis, University of Oslo, 0316 Oslo, Norway; 4 School of Biology and Center of Excellence in Phylogeny of Living Organisms, University of Tehran, 14155-6455 Tehran, Iran; 5 School of Biological Sciences, University of Portsmouth, Portsmouth PO1 2DY, UK; 6 Institute of Arctic Biology, University of Alaska, Fairbanks AK 99775, USA

**Keywords:** evolutionary rate, G-matrix, integration, macroevolution, microevolution, power relationship

## Abstract

If genetic constraints are important, then rates and direction of evolution should be related to trait evolvability. Here we use recently developed measures of evolvability to test the genetic constraint hypothesis with quantitative genetic data on floral morphology from the Neotropical vine *Dalechampia scandens* (Euphorbiaceae). These measures were compared against rates of evolution and patterns of divergence among 24 populations in two species in the *D. scandens* species complex. We found clear evidence for genetic constraints, particularly among traits that were tightly phenotypically integrated. This relationship between evolvability and evolutionary divergence is puzzling, because the estimated evolvabilities seem too large to constitute real constraints. We suggest that this paradox can be explained by a combination of weak stabilizing selection around moving adaptive optima and small realized evolvabilities relative to the observed additive genetic variance.

## 1. Introduction

Linking macro- to microevolution is one of the fundamental challenges in evolutionary theory. Population and quantitative genetics provide precise predictions for the short-term dynamics of allele frequencies and phenotypes, but how far can these predictions be extrapolated? It is customary to distinguish two extreme positions. The first is the extrapolationist view that macroevolution is microevolution writ large, or simply that macroevolution can be fully understood by use of concepts and parameters from quantitative genetic theory (e.g. [1–6]). The alternative extreme is that macroevolution is decoupled from microevolution in such a way that microevolutionary theory is largely irrelevant, and different conceptual tools must be used when studying the two levels (e.g. [7–10]). Most biologists, including those cited above, would probably agree that the truth is somewhere in between these extremes, but exactly how far microevolutionary models can be extended remains an open question [11].

The research paradigm in evolutionary quantitative genetics initiated by Lande and Arnold (e.g. [12,13]) is a good illustration of the extrapolationist view. The fundamental assumptions of this approach include the view that quantitative genetic parameters such as the additive genetic, or at least the mutational, variance parameters remain stable over long stretches of time, allowing rather simple extrapolations of single-generation responses to selection. On this basis, predictions have been derived for patterns of among-species variation based on a variety of models from life-history theory, sexual selection, behavioural ecology or neutral theory (e.g. [14–16]).

A key test of the macroevolutionary relevance of evolutionary quantitative genetics is to see whether macroevolutionary divergence is influenced by patterns of genetic variation as measured in contemporary populations. If there is no such relationship, then either genetic constraints are not important or they are not captured by the observed patterns of genetic variation. Many studies have asked this question, most concluding qualitatively in support of constraint (see Discussion). However, such studies face substantial conceptual and methodological challenges [17–20].

The most important determinant of phenotypic divergence among populations is likely to be the dynamics of local peaks in the adaptive landscape [3,6,21–23]. Low evolvability will affect the degree of divergence by creating a lag or even precluding populations from tracking moving peaks in a changing environment. Only if evolvabilities are small relative to the rate of change in the adaptive landscape do we expect a real constraint on divergence. Thus, looking for a relationship between evolvability and divergence constitutes a test of the importance of constraints in evolution. In particular, it may help clarify the relevant timescales at which genetic constraints are important and thereby the generality of microevolutionary models.

Here, we use recently developed theory on the measurement of evolutionary potential in a multivariate context [17], and connect this with patterns of divergence by explicit evolutionary models. Our approach enables us to investigate how empirical data fit a range of evolutionary scenarios. We also investigate the effect of integration on divergence by comparing independent trait evolution within two sets of traits differing in their degree of evolutionary integration. To do this, we have estimated G-matrices of floral traits in two distinct, albeit unrecognized, species in the Neotropical *Dalechampia scandens* species complex, which we then compare with among-population divergence in 24 populations (12 each from the two species).

## 2. Theory

### (a) Measuring evolvability

Trait evolvability can be measured as the expected proportional response per generation to linear directional selection of unit strength [17,24,25]. Unit strength of selection is the strength of selection on relative fitness as a trait and is given by a (mean-scaled) selection gradient of unity. We denote this measure as *e*, and from the standard equations of quantitative genetics, we get *e* ≡ Δ*z/β* = *I*_A_, where Δ*z* is the mean-scaled selection response, *β* is the mean-scaled selection gradient and *I*_A_ is the mean-standardized additive genetic variance [25]. A value of *e* of, say, 0.01 means that the expected response per generation per unit directional selection is 1% of the trait mean. In the following, we will drop the conceptual distinction between *e* and *I*_A_, and just use the symbol *e*.

While *e* is a straightforward measure of evolvability for a univariate trait, the measurement of multivariate evolvability is more complicated, because the response to selection may then deviate from the direction of the selection gradient and the evolvability may be different in different directions in phenotype space. Hansen & Houle [17] proposed three measures of multivariate evolvability that we will consider here. These are all computed as functions of a given selection gradient, **β** (a column vector of partial regression coefficients), standardized to unit length and the additive genetic variance matrix, **G**. The ‘respondability’, *r*(**β**) = √(**β**‘**G**^2^**β**) is defined as the expected length of the response vector; the ‘evolvability’, *e*(**β**) = **β**‘**Gβ**, is defined as the expected length of the projection of the response vector on the selection gradient; and the conditional evolvability, *c*(**β**) = (**β**‘**G**^−1^**β**)^−1^, is defined as the expected length of the response vector when the directional selection along **β** has come to a balance with assumed stabilizing selection orthogonal to **β** (*c*(**β**) depends only on the existence and not on the strength of the stabilizing selection [26]). The respondability may be interpreted as the ability to change in response to selection, the evolvability as the ability to change in the direction of selection, and the conditional evolvability as the ability to change in the direction of selection when there is stabilizing selection on the perpendicular directions. All these measures reduce to *e* when only a single trait is concerned.

### (b) Relating evolvability to evolutionary divergence

How standing genetic variation relates to macroevolutionary divergence is an open question. Simple models based on extrapolating constant selection and evolvability show that very large changes can be produced from typical estimates of selection strength and evolvability. For example, the mean trait value expected after *t* generations of constant evolvability, *e*, and selection gradient, *β*, is2.1
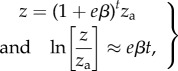
where *z*_a_ is the ancestral trait value. If we combine the median estimate of univariate evolvability for morphological traits from Hansen *et al*. [25] of *e* = 0.1% with the median mean-scaled selection gradient from Hereford *et al*. [27] of *β* = 0.9, we get a doubling of the trait value after 770 generations. Even if selection gradients of this strength are not likely to remain constant over long time periods (e.g. [28,29], but see [30]), this illustrates that typical evolvabilities are not likely to generate macroevolutionary constraints by themselves. The naive expectation from this is that among-species variation is generated by differences in adaptive optima, and that phylogenetic effects have to do with similarities in the optimal states of related species [22,23]. For example, under simple quadratic stabilizing selection, the rate of evolution towards an optimum, measured in generations, would be *βe* = –2*s*(*z* − *θ*)*e*, where *s* is the mean-standardized curvature of the fitness function, *θ* is the optimum, and the distance from the optimum is also measured in units of the trait mean. The time it would take to move half the distance towards the optimum under this model would be *t*_1/2_ = ln2/(2*se*) [23]. With *e* = 0.1% and even a relatively small *s* = 1 (which implies the mean would have to be shifted 45% of the optimum to give *β* = 0.9) it would give *t*_1/2_ ≈ 350 generations, which is again nearly instantaneous on macroevolutionary timescales.

**Figure 1.**
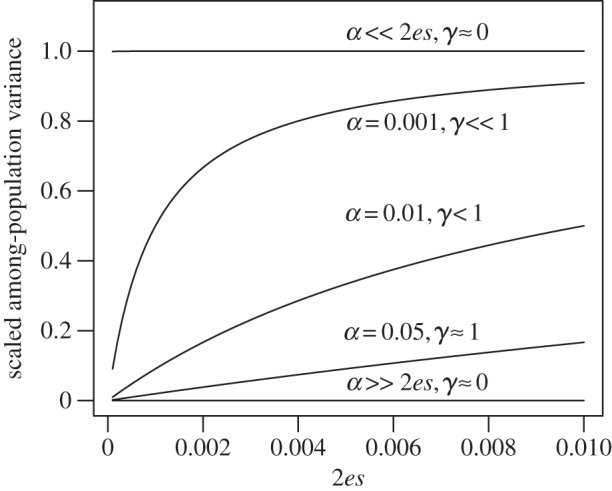
Relationship between among-population variance (var[ln*z*]) and 2*es* given by equation (2.3) for different values of *α*. The approximated scaling exponent between the among-population variance and evolvability on a log–log scale is given by *γ* (the average derivative of the relationship on a log–log scale). The among-population variance is given in units of *V*, the stationary variance of the Ornstein–Uhlenbeck process.

Still, there are many indications of correlations between measures of evolvability and among-population variation (see Discussion). Hence, it is at least possible that evolvabilities, and particularly conditional evolvabilities, of some trait combinations may be small enough to constitute detectable constraints on macroevolutionary timescales. If so, we may find a relationship between measured evolvabilities and among-species variation. Note that proportional changes scale with evolvability, so that we expect among-species variances to scale with the square of evolvability. From equation (2.1), we get2.2
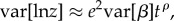
where *ρ* is a constant, and the variance in selection gradients may result from different directions of selection in different populations or from fluctuating selection gradients. For stable differences in direction of selection, we expect scaling with the square of the time since divergence (a scaling exponent of *ρ* = 2), while for fluctuating selection gradients, we expect linear scaling with the time since divergence (a scaling exponent of *ρ* = 1) because the trait mean will then evolve as a Brownian motion. The scaling with the square of evolvability differs from predictions under neutral models, where among-species variance scale linearly with evolvability (e.g. [31–33]). If the trait is tracking a moving optimum, we get different scaling relationships (e.g. [34,35]). Under a simple model of quadratic selection, outlined above (*βe* = −2*s*(*z* − *θ*)*e*), around an optimum, *θ*, that moves according to an Ornstein–Uhlenbeck process (see appendix A in the electronic supplementary material for the analytical derivation), the equilibrium among-species variance in the trait mean becomes2.3
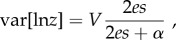
where *V* is the stationary variance of the optimum and *α* is the pull parameter in the Ornstein–Uhlenbeck process. This yields a positive relationship between among-population variance and evolvability that eventually flattens out at an asymptote (figure 1). Note that if 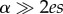, the among-population variance goes to zero. The optima move too fast to be tracked and the populations will experience this as a constant (multiplicative average) optimum. If, on the other hand, the population can track faster than the optimum moves 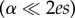, then among-population variance converges on the variance of the optima, and the relationship with evolvability disappears. Hence, stationary fluctuating optima can explain a relationship between evolvability of traits and among-population variance if at least some of the traits have rates of adaptation (2*es*) of the same order of magnitude as the rates of movement in the optimum (*α*). Note also that this common rate would have to be consistent with observed phylogenetic signal in the data. This may require phylogenetic half-lives (*t*_1/2_ = ln2/*α* ≈ ln2/2*es*) on the order of 100 000 generations or more.

These considerations concern the divergence of a one-dimensional trait. Linking evolvability to patterns of multivariate divergence is more complicated, because we rarely have direct information about the multivariate directions of selection or the positions of optima. In most cases, we only have differences between populations to go by. This makes it unclear whether it is the respondability, the evolvability or the conditional evolvability that is most relevant statistic to use. We will assess all of these and test which one gives the best predictions.

## 3. Material and methods

### (a) Study species and blossom traits

**Figure 2.**
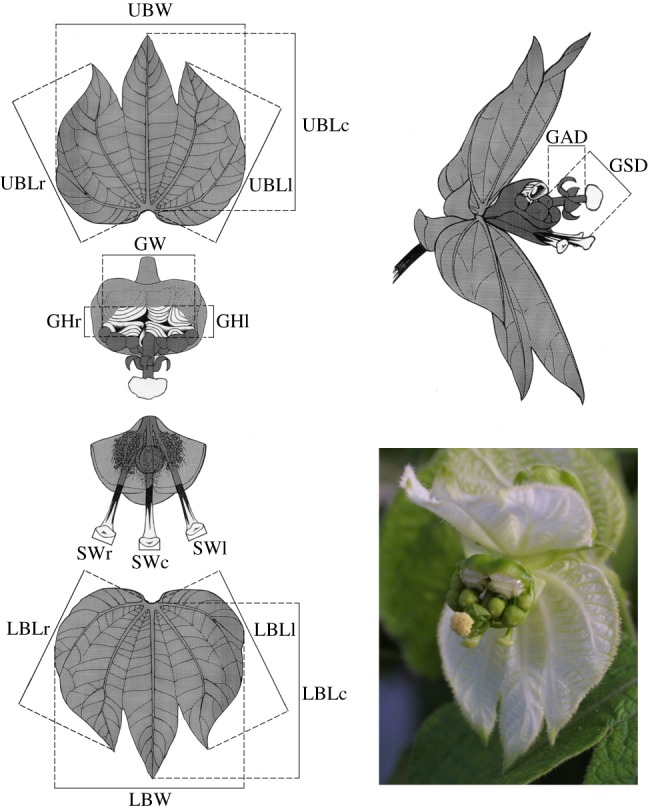
Blossom morphology and measurements (drawing by M. Carlson, photo by C. Pélabon). See table 1 for measurement definitions. (Online version in colour.)

*Dalechampia scandens* L. (Euphorbiaceae) is a Neotropical vine distributed from Mexico to Argentina. Its blossoms (pseudanthial inflorescences) comprise a cluster of three pistillate flowers situated below a cluster of 10 staminate flowers (figure 2) [36,37]. Each female flower contains three ovules so that each blossom can produce a maximum of nine seeds. The flower cluster is subtended by two involucral bracts that may provide a signal to pollinators [38], and may also have a protective role as they close to protect the whole structure at night and during fruit maturation [39]. A gland that produces terpenoid resin is associated with the staminate flowers. The resin varies in colour among *Dalechampia* species and is collected for use in nest construction by various bees in the genera *Eulaema, Eufriesea, Euglossa* (Apoidea: Euglossini), *Hypanthidium* (Megachilidae: Anthidiini) and/or *Trigona* (Apidae: Meliponini). Which bees are attracted depends on characteristics of the blossom and location of the population [40]. Larger glands produce more resin [41], and blossoms with large glands attract larger bees than blossoms with smaller glands [40,42,43]. The efficiency with which different bees transfer pollen is influenced by the distances between the resin gland and the stigma (GSD) and anthers (GAD) [40,42,44].

**Figure 3.**
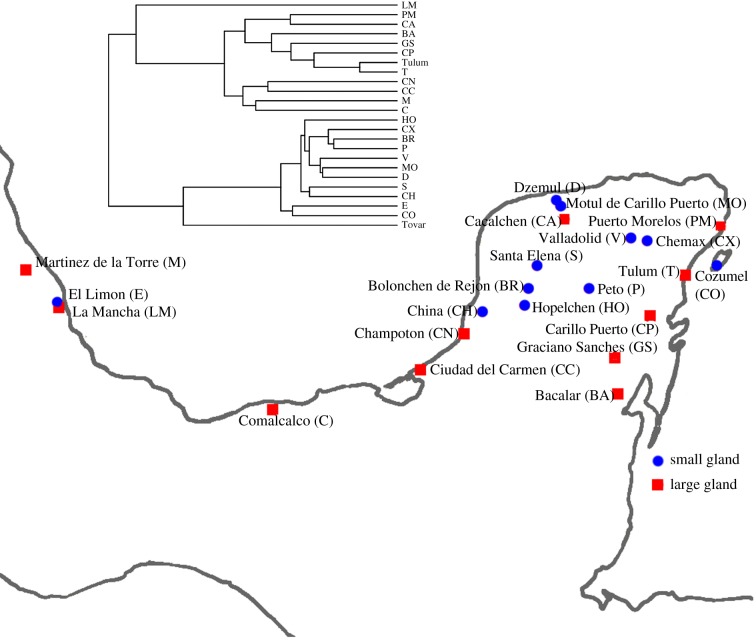
Phylogenetic tree and location of the populations in the Yucatán peninsula and adjacent areas. Populations ‘Tulum’ and ‘T’ are located very closely to each other, but sampled at different times. The population ‘Tovar’ is located in Venezuela. The phylogeny is scaled to unit depth. The phylogeny with branch lengths is deposited in Dryad. (Online version in colour.)

*Dalechampia scandens* contains at least two reproductively isolated groups with overlapping geographical distributions (figure 3). The two ‘species’ differ in blossom size, and particularly in the size of the resin gland. Microsatellite analysis show that they fall out as well-separated groups on the phylogeny (figure 3). Despite many attempts, we have never managed to obtain fertile hybrids between these two species [45,46]. Judging from blossom morphology, the small-glanded species seems to be able to self-pollinate more easily than the large-glanded species.

The morphological measurements used in this study are illustrated in figure 2 and summarized in table 1. One observer (C.P.) measured all plants in the quantitative genetics experiments, and a second observer (G.H.B.) measured all plants in the among-population dataset. Two blossoms were measured for most plants.

**Table 1.** Definition of traits (l, left; r, right); see figure 2 for measurements.

trait	units	abbreviation	definition
functional traits			
gland–anther distance	mm	GAD	GAD
gland–stigma distance	mm	GSD	⅓(GSDl + GSDc + GSDr)
style width	mm	SW	⅓(SWl + SWc + SWr)
gland size	mm	√GA	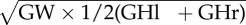
bract size	mm	√BA	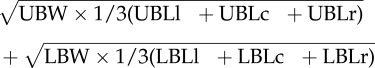
bract traits			
upper-bract-length centre	mm	UBLc	UBLc
upper-bract-length sides	mm	UBLs	1/2 (UBLl + UBLr)
upper-bract width	mm	UBW	UBW
lower-bract-length centre	mm	LBLc	LBLc
lower-bract-length sides	mm	LBLs	1/2 (LBLl + LBLr)
lower-bract width	mm	LBW	LBW

### (b) Quantitative genetics experiments

The plants used in the quantitative genetics analyses were derived from seeds collected in two distinct populations, a large-glanded population near Tulum, Mexico (20°13′ N; 87°26′ W) and a small-glanded population near Tovar, Venezuela (8°21′ N, 71°46′ W). Fruits with seeds were collected from separate individuals in these two populations in 1998. We germinated one seed per maternal family and conducted two separate block diallels in which 12 and nine sets of five parents, in Tulum and Tovar, respectively, were combined into complete 5 × 5 diallels with both reciprocals and selfed crosses. Two individuals were raised from each cross and subsequently measured. The first diallel (Tulum) was conducted between 1999 and 2000 and results from this have been published [24,47,48]. The second diallel (Tovar) was conducted between May 2005 and June 2006. The measurements in the two diallels were similar, but while blossoms with one to three open male flowers were measured in the Tulum diallel, only blossoms with a single open male flower were measured in the Tovar diallel.

### (c) Among-population data

In total, we obtained data on trait means from 24 populations (electronic supplementary material, tables S1–S3), including the two populations on which the quantitative genetics experiments were conducted (see above). The measured plants in the remaining 22 populations were from fruits with seeds sampled from roadsides in Mexico (states of Veracruz, Tabasco, Campeche, Yucatán and Quitana Roo; see the electronic supplementary material, table S1 for exact locations) during the autumn of 2007. All plants were grown in the same greenhouse in Trondheim (Norway) during the same time period. Sample sizes ranged from one to 33 (median 12) plants per population (electronic supplementary material, table S2).

We constructed a neighbour-joining tree (figure 3) based on the genetic-distance measure *D*_A_ [49] using 70 microsatellite markers developed for *D. scandens* [50]. The genetic distances had a perfect tree structure, suggesting limited gene flow between populations. We therefore interpret the genetic distances as reflecting time since divergence. The program Populations 1.2.31 was used to estimate this tree, which we interpret as a phylogeny. See appendix B in the electronic supplementary material for details.

### (d) Data analysis

All analyses were conducted on two sets of traits (table 1). The first set included five functionally related traits: gland–anther distance, gland–stigma distance, style width, gland size (square root of gland area) and bract size (square root of bract area). The second set included six morphologically integrated bract traits: upper-bract-length centre, upper-bract-length sides, upper-bract width, lower-bract-length centre, lower-bract-length sides and lower-bract width.

#### (i) Within-population variation

For the quantitative genetic experiments, we fitted mixed models with the R package MCMCglmm [51]
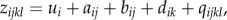
where *z* is the trait value, *u* is the trait mean, *a* is the breeding value, *b* is the non-genetic plant-level effect, *d* is the measurement-date effect and *q* is the residual within-plant effect. The subscripts, *i*, *j*, *k* and *l* represent trait type, plant, day and blossom, respectively. The random effects are assumed to be distributed as **a** ∼ *N*(**0**, **G** ⊗ **A**), **b** ∼ *N*(**0**, **B** ⊗ **I**), **d** ∼ *N*(**0**, **F** ⊗ **I**) and **q** ∼ *N*(**0**, **E** ⊗ **I**), where **A** is the additive relatedness matrix, **I** is the identity matrix and ⊗ is the Kronecker product. The model estimates the additive genetic variance matrix **G**, the among-plant environmental variance matrix **B**, the among-date variance matrix **F** and the residual variance matrix **E**. The traits were mean standardized before the analyses to obtain mean-standardized variance matrices. The complete posterior distributions of all G-matrices are reported in the electronic supplementary material, tables S4–S7.

As priors for the Bayesian mixed models (MCMCglmm), we used zero-mean normal distributions with very large variances (10^8^) for the fixed effects, half-Cauchy distributions with scale parameter 20 [52] for the variance components, and inverse-Wishart distributions with parameters **V** and *n* for the residuals. The matrix parameter **V** was a crude guess based on the phenotypic variance matrix, and the value of *n* was set to *x* − 0.998, where *x* is number of traits in the analysis [51]. The models were robust against changes in the priors, but note that the influence of these priors on functions of variance components is not well understood (JD Hadfield 2014, personal communication). These models ran for 1 100 000 iterations, with a burn-in phase of 100 000 and a thinning interval of 1000. Visual inspection of trace plots showed that the posterior distributions had good convergence and mixing of chains. The autocorrelation was less than 0.1 per sampled iteration for almost all chains.

#### (ii) Evolvability measures

To calculate evolvability measures from the estimated G-matrices, we followed the approach of Hansen & Houle [17]. We have implemented functions to calculate these measures in the R package evolvability (see appendix C, electronic supplementary material). The measures *e*(**β**), *r*(**β**) and *c*(**β**) are explained in the theory section. We also use a measure of evolutionary integration, *i*(**β**) = 1 − *c*(**β**)/*e*(**β**), that measures the fraction of additive genetic variance bound up in the other traits. This integration index varies between zero, no integration, and one complete integration. To calculate the average, the minimum and the maximum evolvability (*e*_mean_, *e*_min_ and *e*_max_) for each G-matrix, we used the average, the minimum and the maximum of the eigenvalues. The minimum and maximum evolvability correspond to the evolvability in the directions of the smallest and largest eigenvector of **G**, while the average evolvability corresponds to the expected evolvability in a random direction. To calculate the average respondability, conditional evolvability and integration (*r*_mean_, *c*_mean_ and *i*_mean_) we took an average over 1000 random selection gradients uniformly distributed on the unit sphere for *r*(**β**), *c*(**β**) and *i*(**β**) instead of using the analytical approximations in Hansen & Houle ([17], see also [53]). Note that some of these measures are biased due to estimation error in the estimated matrices. For example, the largest eigenvalue is overestimated and the lowest eigenvalue is underestimated. The mean of the eigenvalues is not biased, however.

To generate the set of random selection gradients uniformly distributed in *k* dimensions, we used the function randomBeta in the evolvability R-package. This function samples each element of each vector (selection gradient) independently from a zero-mean Gaussian distribution with unit variance, and subsequently normalizes each vector to unit length.

For direct comparison of G-matrices, we calculated the squared correlation coefficient, *R*^2^, between evolvabilities, *e*(**β**), *r*(**β**) and *c*(**β**), computed along 1000 random selection gradients. These estimates of *R*^2^ describe the amount of variance across directions in the evolvability parameter of one G-matrix that can be explained by the same parameter from another G-matrix. All measures were calculated at each iteration of the posterior distribution of the G-matrices to include uncertainty.

#### (iii) Among-population variation

We estimated the among-population variance using phylogenetic mixed models [54,55]. Because of the small number of populations considered, we fitted only univariate models for the among-population variance. We used the natural logarithm of the trait values in the analysis because variances on this scale are directly comparable to evolvability measures. The small- and large-glanded populations were analysed separately. The phylogenetic mixed models were specified as
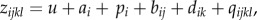
where *z* is the trait value, *u* is the trait mean, *a* is the phylogenetic effect, *p* is the residual population effect, *b* is the plant-level effect, *d* is the measurement-date effect and *q* is the residual within-plant effect. The subscripts, *i*, *j*, *k* and *l* represent population, plant, day and blossom, respectively. Random effects were assumed to be identically independently normally distributed, with the exception of the phylogenetic effects for which we allowed phylogentic correlations as 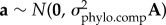, where **A** is the phylogenetic relatedness matrix composed of shared branch lengths between populations. We also fitted models in which the residual population effects (*p*) were excluded, which gave us an estimate of the evolutionary rates 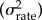, that is, the phylogenetically corrected among-population variance. This is the parameter we used when comparing population divergence and evolvability. Evolutionary rates are measured as increase in variance per unit of time, here the length of the phylogeny, among taxa evolving independently as if by a Brownian motion. The phylogenetic mixed models were fitted using the R-package MCMCglmm with the same specifications as in the genetic models.

#### (iv) Population and species divergence

To understand whether population differentiation had happened along lines of high evolvability, we compared evolutionary rates 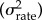 and evolvability (*e*, *r* and *c*) for all traits and for 1000 random directions (unit-length selection gradients). The different evolutionary models predict different scaling relationships between evolvability and evolutionary divergence (figure 1). These scaling relationships were investigated by plotting the log evolutionary rates against log evolvability and comparing this to isometry (a scaling exponent of 1). We did not estimate the scaling exponents directly because a rigorous statistical method for this has not yet been developed. The direction of the vector of species divergence (**β**_species_) was compared to the range of evolvabilities of the G-matrices to see whether this direction had high or low evolvability. Each element of **β**_species_ was calculated by subtracting the average trait values (on the natural log scale) of the small species from the average trait values of the large species estimated in the phylogenetic mixed models, and dividing by the norm of this vector for standardizing to unit length. Uncertainty was assessed by evaluating the complete posterior distributions.

Morphological integration may constrain the independent evolution of individual traits [56]. We investigated the effect of integration by comparing *i*(**β**) with the fraction of independent among-population variance (the ratio of conditional variance among populations over the total among-population variance). Because of statistical power, we were only able to estimate two-dimensional among-population variance matrices using the phylogenetic mixed model described above (not including the independent population effects, *p*). We therefore only compared the pairwise combinations of all traits for autonomy and fraction of independent among-population variance. All analyses were done in R v. 2.15.2 [57].

## 3. Results

### (a) Patterns of evolvability

Average functional-trait evolvabilities were *e* = 0.55% and 0.23% for the small-glanded and large-glanded species, respectively (table 2). These averages are well within the normal range, but larger than the median *e* = 0.09% for linear traits reported in a recent compilation [25]. The traits ranked similarly regarding evolvability in the two species, with the striking exception of gland–anther distance being the most evolvable (*e* = 0.85%) in the small-glanded species, but the least evolvable in the large-glanded species (*e* = 0.06%; table 3). Much of the variation in trait evolvabilities was probably due to estimation error. The functional traits were not very integrated, with mean integration across random directions of *i* = 0.31 and 0.44 for the small- and the large-glanded species, respectively (table 2). This means that conditional evolvabilities on average were as high as 69% and 56% of the unconditional evolvabilities. Hence, most combinations of functional traits had a high degree of independent evolutionary potential.

**Table 2.** Means of evolvability measures (*e*, *r* and *c* in % of trait mean) and integration (*i* in proportions) over uniformly distributed random directions in the G-matrices. The maximum and minimum values of *e* are given by the highest and lowest eigenvalue, respectively. Estimates are posterior medians with 95% highest posterior density interval in parentheses.

	functional traits		bract traits	
	Tovar	Tulum	Tovar	Tulum
*e*_mean_	0.55 (0.47, 0.66)	0.23 (0.17, 0.30)	0.40 (0.30, 0.49)	0.27 (0.20, 0.35)
*e*_min_	0.140 (0.097, 0.182)	0.037 (0.001, 0.069)	0.003 (0.001, 0.005)	0.002 (0.001, 0.004)
*e*_max_	1.28 (0.99, 1.68)	0.64 (0.36, 0.90)	2.12 (1.64, 2.71)	1.47 (1.07, 2.00)
*r*_mean_	0.64 (0.52, 0.77)	0.29 (0.19, 0.38)	0.76 (0.60, 0.97)	0.52 (0.39, 0.71)
*c*_mean_	0.38 (0.31, 0.44)	0.12 (0.06, 0.17)	0.016 (0.007, 0.023)	0.009 (0.004, 0.014)
*i*_mean_	0.31 (0.24, 0.40)	0.44 (0.27, 0.70)	0.91 (0.87, 0.95)	0.92 (0.88, 0.96)

**Table 3.** Functional-trait means (*z*_mean_ in mm) with standard error, and variance components with 95% highest posterior density interval of the quantitative genetic analyses for the five functional traits. The variance components, evolvability (*e*), among-plant environmental variance 
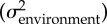
, among-measurement-date variance 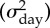 and among-blossom (residual) variance 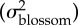 are mean standardized and multiplied by 100.

	GAD	GSD	SW	√GA	√BA
Tovar (a small-glanded population)					
*z*_mean_	3.22 ± 0.08	5.47 ± 0.13	1.13 ± 0.02	4.15 ± 0.06	37.94 ± 0.60
*e*	0.85 (0.65, 1.09)	0.78 (0.50, 1.11)	0.50 (0.33, 0.64)	0.22 (0.16, 0.33)	0.32 (0.26, 0.46)
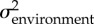	≈0 (0.00, 0.07)	≈0 (0.00, 0.26)	≈0 (0.00, 0.07)	≈0 (0.00, 0.04)	≈0 (0.00, 0.02)
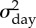	0.35 (0.20, 0.58)	0.20 (0.08, 0.34)	0.21 (0.11, 0.33)	0.10 (0.04, 0.16)	0.09 (0.05, 0.15)
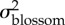	0.94 (0.83, 1.09)	1.34 (1.14, 1.51)	0.77 (0.66, 0.88)	0.54 (0.47, 0.61)	0.46 (0.40, 0.52)
Tulum (a large-glanded population)					
*z*_mean_	4.63 ± 0.04	4.61 ± 0.05	1.36 ± 0.01	4.37 ± 0.04	38.19 ± 0.34
*e*	0.06 (0.01, 0.13)	0.34 (0.23, 0.51)	0.31 (0.20, 0.45)	0.19 (0.08, 0.28)	0.21 (0.16, 0.31)
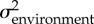	≈0 (0.00, 0.08)	≈0 (0.00, 0.04)	≈0 (0.00, 0.04)	≈0 (0.00, 0.06)	≈0 (0.00, 0.03)
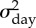	0.21 (0.16, 0.38)	0.16 (0.10, 0.29)	0.29 (0.23, 0.51)	0.25 (0.16, 0.41)	0.17 (0.10, 0.25)
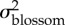	0.92 (0.85, 1.06)	1.20 (1.07, 1.33)	0.88 (0.78, 1.01)	1.26 (1.17, 1.45)	0.56 (0.52, 0.65)

Bract traits had average evolvabilities of *e* = 0.40% and 0.27% for the small- and large-glanded species, respectively (table 2). These averages were similar to those of the functional traits, but the bract traits were much more integrated and had more equal evolvabilities (tables 2 and 4). The high average integration of *i* = 0.91 and 0.92 means that conditional evolvabilities would be very low for most combinations of bract traits. The averages of *c* ≈ 0.01% indicate a potential change of only a hundredth of a per cent per generation under unit selection, and the minimum evolvabilities were almost an order of magnitude below this. Note, however, that bract traits may still be highly evolvable along a few directions. Indeed, the maximum evolvabilities, which equal the maximum conditional evolvabilities, were 2.1% and 1.5% for the small- and large-glanded species, respectively (table 2).

**Table 4.** Bract-trait means (*z*_mean_ in mm) with standard error and variance components with 95% highest posterior density interval of the quantitative genetic analyses for the six bract traits. See table 3 for further explanation.

	UBLc	UBLs	UBW	LBLc	LBLs	LBW
Tovar (a small-glanded population)						
*z*_mean_	18.97 ± 0.31	16.54 ± 0.26	19.49 ± 0.33	21.24 ± 0.38	18.27 ± 0.32	19.99 ± 0.34
*e*	0.35 (0.28, 0.46)	0.32 (0.27, 0.44)	0.37(0.27, 0.51)	0.45 (0.34, 0.56)	0.46 (0.35, 0.55)	0.36 (0.27, 0.52)
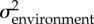	≈0 (0.00, 0.01)	≈0 (0.00, 0.01)	≈0 (0.00, 0.03)	≈0 (0.00, 0.01)	≈0 (0.00, 0.01)	≈0 (0.00, 0.03)
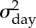	0.05 (0.02, 0.10)	0.04 (0.01, 0.08)	0.09 (0.06, 0.20)	0.042 (0.01, 0.09)	0.040 (0.01, 0.08)	0.13 (0.06, 0.22)
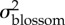	0.45 (0.38, 0.50)	0.40 (0.36, 0.46)	0.55 (0.47, 0.62)	0.56 (0.52, 0.67)	0.51 (0.44, 0.57)	0.73 (0.63, 0.81)
Tulum (a large-glanded population)						
*z*_mean_	18.88 ± 0.17	16.63 ± 0.13	20.32 ± 0.18	20.52 ± 0.20	17.38 ± 0.16	20.49 ± 0.22
*e*	0.26 (0.18, 0.34)	0.18 (0.13, 0.26)	0.24 (0.18, 0.34)	0.28 (0.20, 0.41)	0.25 (0.17, 0.35)	0.31 (0.21, 0.44)
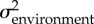	≈0 (0.00, 0.01)	≈0 (0.00, 0.01)	≈0 (0.00, 0.01)	≈0 (0.00, 0.01)	≈0 (0.00, 0.01)	≈0 (0.00, 0.03)
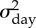	0.18 (0.11, 0.32)	0.12 (0.06, 0.21)	0.14 (0.08, 0.23)	0.21 (0.11, 0.31)	0.15 (0.09, 0.27)	0.30 (0.19, 0.46)
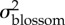	0.58 (0.53, 0.65)	0.60 (0.53, 0.66)	0.61 (0.55, 0.68)	0.73 (0.66, 0.83)	0.69 (0.62, 0.78)	0.87 (0.76, 0.96)

Although the small-glanded species was roughly twice as evolvable as the large-glanded species, their G-matrices were qualitatively similar in the general patterns and levels of integration. There were a lot of particular differences, however, and, for the functional traits, the evolvability measures along different directions were poorly correlated between the matrices (*R*^2^ = 12%, 12%, 7% for *r*, *e* and *c*, respectively). The bract-trait matrices were more consistent (*R*^2^ = 96%, 97%, 36% for *r*, *e* and *c*, respectively).

In both small- and large-glanded populations, the patterns of respondability were similar to the patterns of evolvability. We will therefore not discuss respondability further (but see the electronic supplementary material). As for non-genetic variance components, we note that there were large components of temporal variance (‘day’) and that most of the residual variance was among individual blossoms and not among plants.

### (b) Patterns of evolutionary rates

The phylogenetic structure explained a substantial amount of variation in all the measured traits, with phylogenetic heritabilities [54] ranging from approximately 0.5 to 0.8 (tables 5 and 6). For this reason, we focus on estimated evolutionary rates along the phylogeny instead of the raw among-population variances. These evolutionary rates are the phylogenetically corrected among-population variances 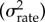. We focus on the square roots of these variances (i.e. CV_rate_) because they scale isometrically with the evolvabilities under linear selection (see equations (2.1) and (2.2)). The mean-scaled variance accumulated over the length of the phylogeny was around 0.02 (ln mm)^2^ for all traits; this equals a CV_rate_ of around 14%. The exception was bract traits in the small-glanded species, which had rates of evolution an order of magnitude lower than the other traits (tables 5 and 6).

**Table 5.** Variance components of the phylogenetic analyses for the five functional traits. The evolutionary rates 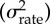 and the phylogenetic variance 
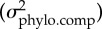
 have units of 100 × (ln mm)^2^/*t*, where *t* is the length of the phylogeny (this is equal to the mean-scaled variance accumulated over the length of the phylogeny in %). The phylogenetic heritability, 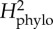, is given by 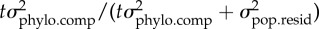. The other variance components, the population-residual variance 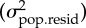, the among-day variance 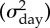, the among-plant variance 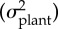 and the within-plant variance (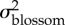), have units of 100 × (ln mm)^2^. Estimates are posterior medians with 95% highest posterior density interval in parentheses.

	GAD	GSD	SW	√GA	√BA
phylogenetic analysis for the small-glanded populations					
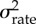	1.40 (0.16, 3.04)	3.77 (0.66, 8.16)	2.97 (0.36, 6.76)	1.15 (0.17, 2.61)	0.15 (0.00, 0.49)
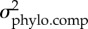	0.85 (0.00, 2.47)	1.90 (0.00, 6.10)	1.32 (0.00, 4.49)	0.92 (0.00, 2.67)	0.13 (0.00, 0.52)
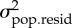	0.44 (0.00, 1.37)	1.34 (0.00, 3.63)	1.04 (0.00, 2.87)	0.30 (0.00, 1.10)	0.08 (0.00, 0.27)
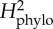	0.58 (0.01, 1.00)	0.51 (0.01, 1.00)	0.47 (0.10, 1.00)	0.71 (0.05, 1.00)	0.55 (0.01, 1.00)
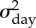	0.13 (0.00, 0.39)	0.13 (0.00, 0.44)	0.26 (0.00, 0.68)	0.19 (0.00, 0.53)	0.08 (0.00, 0.24)
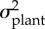	0.17 (0.00, 0.48)	0.79 (0.00, 1.56)	0.34 (0.00, 0.82)	0.20 (0.00, 0.64)	0.11 (0.00, 0.34)
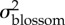	1.88 (1.40, 2.42)	3.68 (2.70, 4.71)	2.16 (1.56, 2.81)	2.15 (1.58, 2.71)	1.23 (0.93, 1.59)
phylogenetic analysis for the large-glanded populations					
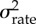	1.44 (0.19, 3.22)	1.67 (0.12, 3.72)	1.89 (0.42, 4.31)	2.24 (0.60, 4.82)	1.54 (0.36, 3.23)
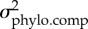	0.95 (0.00, 2.93)	1.11 (0.00, 3.21)	1.45 (0.00, 3.69)	1.12 (0.00, 3.57)	1.01 (0.00, 2.71)
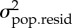	0.54 (0.00, 1.74)	0.59 (0.00, 1.91)	0.52 (0.00, 1.89)	0.73 (0.00, 1.96)	0.49 (0.00, 1.63)
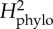	0.58 (0.01, 1.00)	0.60 (0.01, 1.00)	0.73 (0.10, 1.00)	0.53 (0.05, 1.00)	0.64 (0.01, 1.00)
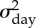	0.35 (0.00, 0.77)	0.10 (0.00, 0.29)	0.25 (0.00, 0.60)	0.07 (0.00, 0.22)	0.10 (0.00, 0.26)
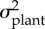	0.76 (0.21, 1.46)	0.54 (0.05, 1.04)	0.50 (0.00, 1.02)	0.07 (0.00, 0.24)	0.16 (0.00, 0.38)
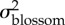	1.70 (1.22, 2.21)	1.70 (1.28, 2.15)	1.79 (1.38, 2.31)	1.25 (0.96, 1.54)	1.04 (0.77, 1.32)

**Table 6.** Variance components of the phylogenetic analyses for the six bract traits. See table 5 for further explanation.

	UBLc	UBLs	UBW	LBLc	LBLs	LBW
phylogenetic analysis for the small-glanded populations						
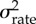	0.14 (0.00, 0.66)	0.10 (0.00, 0.44)	0.29 (0.00, 0.99)	0.34 (0.00, 1.01)	0.26 (0.00, 0.83)	0.12 (0.00, 0.78)
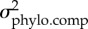	0.11 (0.00, 0.70)	0.08 (0.00, 0.44)	0.14 (0.00, 0.88)	0.20 (0.00, 0.91)	0.18 (0.00, 0.74)	0.10 (0.00, 0.68)
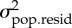	0.05 (0.00, 0.31)	0.02 (0.00, 0.211)	0.10 (0.00, 0.51)	0.10 (0.00, 0.51)	0.06 (0.00, 0.36)	0.06 (0.00, 0.46)
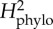	0.71 (0.01, 1.00)	0.78 (0.03, 1.0)	0.55 (0.01, 1.00)	0.67 (0.01, 1.00)	0.76 (0.02, 1.00)	0.63 (0.01, 1.00)
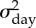	0.05 (0.00, 0.24)	0.04 (0.00, 0.19)	0.09 (0.00, 0.34)	0.03 (0.00, 0.19)	0.06 (0.00, 0.23)	0.07 (0.00, 0.32)
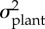	0.03 (0.00, 0.22)	0.06 (0.00, 0.25)	0.10 (0.00, 0.41)	0.09 (0.00, 0.45)	0.04 (0.00, 0.24)	0.20 (0.00, 0.63)
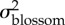	1.29 (0.97, 1.62)	1.07 (0.79, 1.34)	1.53 (1.15, 2.00)	1.54 (1.18, 1.98)	1.11 (0.82, 1.38)	1.52 (1.11, 2.00)
phylogenetic analysis for the large-glanded populations						
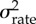	1.93 (0.59, 4.49)	1.76 (0.42, 4.21)	1.16 (0.19, 3.22)	1.63 (0.39, 4.23)	1.45 (0.43, 3.63)	1.47 (0.36, 4.04)
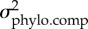	1.30 (0.00, 4.32)	1.01 (0.00, 3.38)	0.60 (0.00, 2.36)	0.98 (0.00, 3.53)	0.93 (0.00, 2.86)	0.90 (0.00, 3.34)
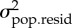	0.37 (0.00, 2.14)	0.44 (0.00, 1.88)	0.30 (0.00, 1.30)	0.38 (0.00, 1.87)	0.29 (0.00, 1.41)	0.34 (0.00, 1.81)
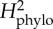	0.80 (0.03, 1.00)	0.71 (0.01, 1.00)	0.66 (0.01, 1.00)	0.72 (0.02, 1.00)	0.78 (0.04, 1.00)	0.75 (0.03, 1.00)
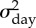	0.11 (0.00, 0.39)	0.13 (0.00, 0.42)	0.07 (0.00, 0.29)	0.07 (0.00, 0.28)	0.08 (0.00, 0.25)	0.09 (0.00, 0.29)
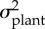	0.14 (0.00, 0.45)	0.16 (0.00, 0.48)	0.14 (0.00, 0.39)	0.32 (0.05, 0.65)	0.20 (0.00, 0.44)	0.12 (0.00, 0.38)
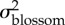	1.37 (1.02, 1.76)	1.31 (0.95, 1.68)	1.10 (0.79, 1.37)	1.19 (0.89, 1.57)	1.06 (0.78, 1.40)	1.24 (0.92, 1.55)

### (c) Relationship between evolvability and divergence

**Figure 4.**
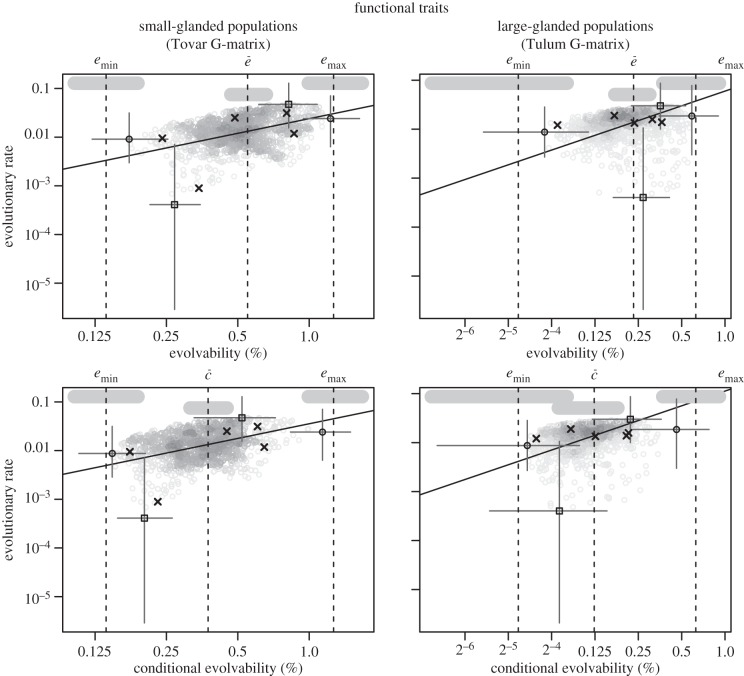
Scaling relationship between evolutionary rate 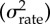 and evolvability for the functional traits. Grey circles represent 1000 uniformly distributed random directions (selection gradients). The solid line indicates the isometric relationship (a slope of 1) passing through the mean of the random directions. Crosses are the measured traits, squares are the directions with highest or lowest trait divergence, and black circles are the directions with highest and lowest evolvability out of the 1000 random directions. The circles, crosses and squares are the modes from the posterior distributions and the grey lines give the 95% highest posterior density intervals. The vertical dotted lines are the posterior modes for the parameters named above each plot, and the vertical thick grey bars are their 95% highest posterior density intervals (table 2). The differences between minimum and maximum evolvability and the lowest and highest evolvability of the random directions are due partly to sampling error and partly to bias in the estimates of *e*_min_ and *e*_max_.

**Figure 5.**
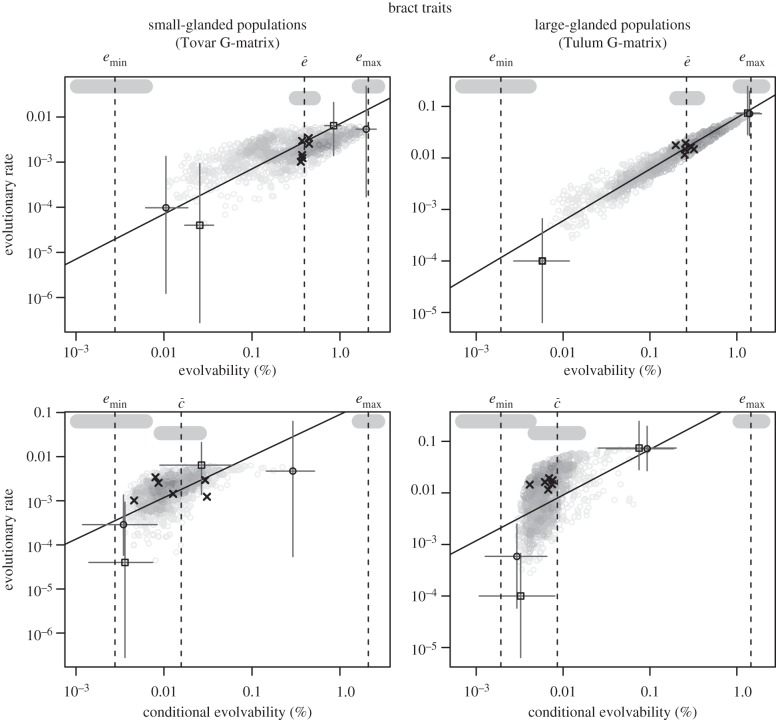
Scaling relationship between evolutionary rates 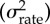 and evolvability for the bract traits. See figure 4 for explanation of symbols.

Note first that the rates of evolution were very small relative to the estimated evolvabilities. With our estimated evolvabilities, changes of this magnitude could be produced by natural selection over just a few generations. We do not have direct information about the absolute age of the phylogeny, but because the deepest split in the phylogeny is between species, we regard it as likely that the populations within each species have been separated by hundreds of thousands of years. Hence, there is no obvious reason to expect an influence of genetic constraints. However, table 7 shows that there was high evolvability in the direction of the species divergence, and figures 4 and 5 show that there was a relationship between evolvability and population divergence, with populations having diverged more in directions of high evolvability. This holds true for conditional and unconditional evolvability in both functional traits and bract traits in both species. Note in particular the strong, nearly isometric, relationship between evolvability and evolutionary rate in the bract traits shown in figure 5. These relationships are not just due to a vague general match between the G-matrices and the among-population variance matrices. If we swap the G-matrices, and try using the small-glanded G-matrix to predict population divergence in the large-glanded species or vice versa, the relationships disappear for the functional traits (electronic supplementary material, figures S1 and S2). This underscores that **G** can change over time, and thereby changing the predictions of among-population divergence. In general, divergence is best predicted by evolvability, less well by conditional evolvability and hardly at all by respondability (see the electronic supplementary material, figures S3 and S4 for results on respondability).

**Table 7.** Evolvability measures (*e*(**β**), *r*(**β**) and *c*(**β**) in %) along the vector between the species means (**β**_species_) in the different G-matrices.^a^ Estimates are posterior medians with 95% highest posterior density interval in parentheses.

	functional traits		bract traits	
	Tovar	Tulum	Tovar	Tulum
*e*(**β**_species_)	0.97 (0.66, 1.33)	0.41 (0.18, 0.65)	1.92 (1.45, 2.55)	1.32 (0.88, 1.79)
*r*(**β**_species_)	1.05 (0.73, 1.39)	0.49 (0.25, 0.75)	2.00 (1.52, 2.59)	1.38 (0.96, 1.87)
*c*(**β**_species_)	0.66 (0.35, 1.03)	0.14 (0.01, 0.29)	0.13 (0.00, 0.57)	0.07 (0.01, 0.30)

^a^The posterior medians of the unit length vectors of species differences are ***β***_species_ = 0.585 × lnGAD + 0.345 × lnGSD + 0.429 × lnSW + 0.479 × ln√GA + 0.298 × ln√BA for the functional traits and ***β***_species_ = 0.362 × lnUBLc + 0.427 × lnUBLs + 0.435 × lnUBW + 0.382 × lnLBLc + 0.387 × lnLBLs + 0.371 × lnLBW for the bract traits.

**Figure 6.**
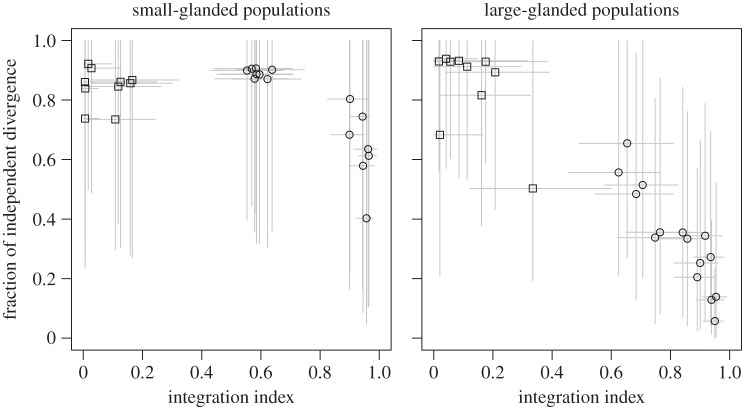
Relationship between pairwise evolutionary integration and independent divergence expressed as the fraction of among-population variance in the focal trait that is independent of the other trait. Each point is a pairwise comparison of two traits. Circles (bract traits) and squares (functional traits) are posterior medians and the grey lines give the 95% highest posterior density interval.

General estimates of conditional evolvability involving many traits are error prone, and it is not surprising that the relationship with among-population variation was noisy. We can get more precise estimates by conditioning single traits on each other. This is asking how much one trait is likely to constrain the evolution of another trait. We did this for all pairwise combinations of traits and then tested whether the integration (*i* = 1−conditional evolvability/evolvability) between pairs of traits predicts the independent evolution of the traits. There was a strong negative relationship between integration and independent evolution for the large-glanded species, but a less clear relationship for the small-glanded species (figure 6).

## 4. Discussion

Our results are consistent with genetic constraints on trait divergence. Both the direction with highest among-population divergence and the direction of divergence between the two species had high evolvabilities compared to average directions (figures 4 and 5; table 7). This was true for both the functionally related pollination traits and for the morphologically related bract traits. The two trait groups differed strikingly in their patterns of integration, however, with the morphologically related bract traits being much more integrated, with much lower conditional evolvabilities.

### (a) Modes of evolution in *Dalechampia scandens*

*Dalechampia* blossom morphology is under direct selection from both pollinators and seed predators [38,58–60]. Comparative analyses show that the fitness optima of *Dalechampia* blossom traits are influenced by several factors, including bee community composition, availability of other resin sources for the bees, presence of other *Dalechampia* species and energetic constraints [40,42,44,61], but only a small part of the interpopulation variation has been explained by models of selective factors [44,61]. This may be due to the crude way the selective factors have been modelled, or it may be due to genetic constraints. Previous studies have shown that pleiotropic constraints can be important in the evolution of blossom traits in *D. scandens* [47,62] and in *Dalechampia* in general [63]. This is supported by this study.

The scaling exponent between evolvability and evolutionary rates for the functional traits was clearly below one for the large-glanded populations and closer to one for the small-glanded populations (figure 4). Such scaling relationships are consistent with models of moving optima in which the population means can almost keep pace with their optima (*α* < 2*es*, figure 1). The similarity in the scaling relationship for unconditional and conditional evolvability makes it hard to judge if stabilizing selection has constrained evolution in certain directions or not.

Among the functional traits, bract size in the small-glanded populations stands out. This trait has a similar amount of additive genetic variance as the other functional traits, but much less among-population variance. This suggests that there is less dispersion of optima for this trait. This pattern is consistent across all bract traits in the small-glanded populations.

The relatively tight scaling relationship, with a scaling exponent below one, between evolutionary rate and evolvability for the bract traits in the small-glanded populations (figure 5) indicates that the population means lag behind their moving optima (*α* ≈ 2*es*, figure 1). With an evolvability of say 0.1%, and moderately weak stabilizing selection (e.g. *s* = 1), the value of *α* will be too large to be consistent with the observation of phylogenetic signal (*α* ≈ 2*es* = 0.001 gives *t*_½_ ≈ 700 generations). For very weak selection (e.g. *s* = 0.01), however, this model may be plausible (*α* ≈ 2*es* = 0.00001 gives *t*_½_ ≈ 70 000 generations). The tight isometric relationship observed for the large-glanded populations (figure 5) can also be consistent with the same model, but the stabilizing selection needs to be even weaker, because *α* would need to be of the order of 10*es* to be consistent with the isometric relationship (figure 1). Such an isometric relationship is also consistent with models of neutral evolution, but the ratio of among-population variance to evolvability, which equals the ratio of generations to effective population size under drift, is orders of magnitude too small.

The main difference between functional and bract traits was their degree of evolutionary integration. This had a strong effect on the relationship between **G** and divergence in both the small- and large-glanded species. The effect of integration on evolution was also reflected in the correspondence between the integration index and independent evolution of the traits in the large-glanded populations (figure 6). This reinforces previous results indicating correlated evolution among blossom traits in *D. scandens* [47,62]. Note, however, that several traits achieved independent evolution despite a high level of integration in the small-glanded populations.

### (b) The paradoxical relationship between **G** and divergence

Taken at face value our evolvability estimates and even most of our conditional-evolvability estimates predict very rapid evolution at macroevolutionary timescales. Yet, we also find clear evidence for a relationship between evolvabilities and patterns of evolution indicating that genetic constraints may be important. How can these two results be reconciled? It is not that this finding is unique to our study. We know of many studies reporting a relationship between patterns of genetic variation and population divergence or evolutionary rates [17,24,64–80] and also many studies reporting a relationship between phenotypic variation and divergence [69,81–92]. The macroevolutionary relevance of evolvability is not that clear cut, however, and some studies have failed to find a relationship and concluded that genetic constraints are not important for divergence [33,84,85,93–100]. Before generalizing from this body of work it is important to realize that there are many unsolved methodological problems stemming both from the difficulties of constructing quantitative measures of constraints in absence of a realistic quantitative theory of macroevolutionary change on a wide range of timescales, and from statistical difficulties with achieving reasonably accurate estimates of **G**. The field is also marred by fundamental measurement/theoretical problems such as use of inappropriate or incommensurate scales, use of theory-free indices and use of statistical significance testing in place of estimation [101]. Hence, the seemingly clear evidence that evolutionary divergence is often constrained must be regarded as tentative. However, we think that the problems will tend to obscure the relationships between evolvability and divergence rather than enhance them. We will briefly go through some such problems and evaluate the studies that have found evidence against the constraint hypothesis in this light.

A common problem, especially with the early studies, is the use of correlation matrices (e.g. [84,94,95,97]). Correlation matrices are poorly suited for investigating the relationship between evolutionary potential and divergence, because they may obscure any order among the measured traits both in amount of divergence and in amount of genetic variation by standardizing all these values to one. The severity of this problem can be seen from the finding that there is no correlation between mean-scaled and variance-scaled additive variances, i.e. no correlation between ‘evolvability and heritability’ [25,102]. Hence, any relationship between ‘evolvability’ and divergence is predicted to be completely randomized after variance standardization such as forming a correlation matrix. We suspect that some studies have failed to find evidence for constraint due to variance standardization.

Schluter's [79] test of constraints based on estimating the angle between the direction of divergence and the largest eigenvalue of **G**, ***g***_max_, is a common denominator across many studies not finding evidence for constraints [85,93,96,97,99,100]. However, this method cannot falsify the constraint hypothesis, because there may be more than one direction with high evolvability [17,66,87,103]. Note that estimating the angle to the directions of several eigenvalues of **G** cannot falsify the constraint hypothesis either, because there may be directions of high genetic variance in between the eigenvectors.

Many studies test constraints by use of various matrix comparison methods to assess the similarity between the G-matrix and among-population D-matrices [33,84,93–95]. Some of these methods such as correlation of matrix elements, often in combination with the overused Mantel tests, have obscure meaning and little statistical justification, but others such as common-principal-component analysis may at least be statistically correct (but see [104]). It is hard to interpret the results from such methods, however, because there is no established theoretical link between the dissimilarity statistics and evolutionary models. Two matrices may be simultaneously similar and dissimilar in many different ways. We do not know how to recognize the influence of genetic constraints in such studies.

Note that *Q*_ST_ − *F*_ST_ studies are not directly relevant for testing the genetic constraint hypothesis, because these are designed to test the null hypothesis of neutral divergence and usually not the relationship between **G** and **D** beyond this ([105–108], but see [98]).

We are left with the two studies of Chenoweth & Blows [98] and Kimmel *et al*. [100] that convincingly demonstrate no constraining effect of **G** on the evolutionary divergence. However, one of these, Chenoweth & Blows [98], is not consistent with the conclusion of a reanalysis of the same data [72] regarding evidence for constraints. We therefore conclude that there is little evidence against and quite a lot of evidence for, the genetic constraint hypothesis, although the methodological problems are also abundant in several of the studies that report a relationship between **G** and divergence. At the same time, quantitative genetic estimates of additive variance generally support high evolvabilities [25,102], and this sends us back to the question of how seemingly high evolvabilities can still be correlated with evolutionary change on million-year timescales.

We consider three possible explanations for the paradoxical relationship between **G** and divergence. First, natural selection may shape within- and between-population variation in a similar manner. This is hard to rule out in the absence of direct information about historical patterns of selection or the movement of fitness optima, but in our opinion theory does not support a strong match between **G** and patterns of selection [109,110]. Also, Blows *et al*. [111] did not find any relationship between a G-matrix and estimated patterns of selection. It is particularly hard to believe that natural selection can explain the paradox when there is strong match between the patterns. For example, in the case of our bract traits in the large-glanded population (figure 5), the distribution of fitness peaks must be almost exactly proportional to **G**.

Second, adaptive optima may move within a restricted area at a pace at which they can be tracked, but not reached. If so, there will be a correlation between evolvability and divergence because populations will track better in directions with high evolvability. This requires, however, that stabilizing selection is very weak; otherwise, the models predict that the population means would perfectly track the optima for any observed levels of evolvability (see equation (2.3)).

The third and last alternative is that realized evolvabilities are much smaller than measured evolvabilities, yet correlated with them. For example, it is possible that a conditional evolvability relative to a set of unmeasured traits under stabilizing selection could be quite small, due to a high degree of pleiotropy, and also correlated with the unconditional evolvabilities, since they both depend on the total variation. Similarly, for macroevolutionary changes, standing genetic variation may be less relevant for long-term response than the supply of ‘mutational evolvability’ based on how much genetic variation is generated each generation, and the mutational and standing evolvabilities are likely correlated. This last alternative is in line with several recent reviews concluding that there is good evidence that genetic constraints are important in evolution [11,18,112,113].

Distinguishing between these and other explanations is an empirical question, but in our opinion, a combination of relatively weak stabilizing selection and small realized evolvabilities is tentatively the most plausible. This can explain why some populations evolve fast on microevolutionary timescales [4,6,114,115], which would not have been possible if realized evolvabilities are always very small, and, at the same time, not completely at odds with the general notion of strong natural selection [18,27,29,116].

## Supplementary Material

Supporting Information

## Supplementary Material

Supplementary Table 4

## Supplementary Material

Supplementary Table 5

## Supplementary Material

Supplementary Table 6

## Supplementary Material

Supplementary Table 7

## Data accessibility

Data available from the Dryad Digital Repository doi:10.5061/dryad.4ff1t.
