# Supplementary material for: Genetic constraints predict evolutionary divergence in Dalechampia blossoms
Source: Philos Trans R Soc Lond B Biol Sci. 2014 Aug 19;369(1649):20130255. doi: 10.1098/rstb.2013.0255 (PMC4084540; doi:10.1098/rstb.2013.0255)
Supplement: Supporting Information [file rstb20130255supp1.pdf]

Supporting Information for

Genetic Constraints Predict Evolutionary Divergence in *Dalechampia* Blossoms

Bolstad, G. H.*, Hansen, T. F., Pélabon C., Falahati-Anbaran, M., Pérez-Barrales, R., Armbruster, W. S.

*To whom correspondence should be addressed. E-mail: geir.h.bolstad@ntnu.no

Tables and figures

Table S1. Name, abbreviation (Abbr.), geographical location given by the state in Mexico and GPS coordinates (WGS84), and meters above sea level (Elevation).

Name	Abbr.	State	Longitude	Longitude	Elevation
Small-glanded populations:					
Cozumel	CO	Quintana Roo	20°22'10" N	86°59'40" W	13
Chemax	CX	Yucatán	20°40'22" N	88°00'25" W	22
Valladolid	V	Yucatán	20°42'31" N	88°15'06" W	31
Peto	P	Yucatán	20°01'34" N	88°52'49" W	38
Motul de Carillo Puerto	MO	Yucatán	21°08'12" N	89°16'45" W	13
Dzemul	D	Yucatán	21°12'35" N	89°19'10" W	0
Santa Elena	S	Yucatán	20°19'55" N	89°37'55" W	37
Bolonchén de Rejón	BR	Campeche	19°59'23" N	89°45'48" W	112
Hopelchén	HO	Campeche	19°46'34" N	89°49'50" W	58
China	CH	Campeche	19°43'25" N	90°24'52" W	3
El Limón	E	Veracruz	19°41'06" N	96°29'48" W	133
Tovar diallel		(Venezuela)	8°21' N	71°46' W	
Large-glanded populations:					
Puerto Morelos	PM	Quintana Roo	20°51'11" N	86°53'43" W	7
Tulúm	T	Quintana Roo	20°12'26" N	87°27'04" W	21
Carillo Puerto	CP	Quintana Roo	19°38'58" N	87°59'00" W	15
Bacalar	BA	Quintana Roo	18°36'40" N	88°26'32" W	27
Graciano Sánches	GS	Quintana Roo	19°08'04" N	88°30'18" W	40
Cacalchén	CA	Yucatán	20°58'01" N	89°13'18" W	13
Champotón	CN	Campeche	19°21'49" N	90°43'06" W	4
Ciudad del Carmen	CC	Campeche	18°56'29" N	91°18'01" W	2
Comalcalco	C	Tabasco	18°21'26" N	93°20'43" W	6
La Mancha	LM	Veracruz	19°37'15" N	96°28'09" W	205
Martinez de la Torre	M	Veracruz	20°05'09" N	97°01'55" W	67
Tulum diallel		Quintana Roo	20°13' N	87°26' W	

Table S2. Summary statistics for the populations and species (averages of the populations). Number of plants and blossoms measured, and trait means and standard error (in mm) for the functional traits. Posterior means with standard errors are obtained from univariate Bayesian mixed models with plant and measurement date as random factors, and population as fixed factor. Separate models were fitted for small- and large-glanded populations. The estimates for the diallel populations were from the multivariate mixed models described in the methods.

Name	#plants	#blossoms	GAD	GSD	SW	√GA	√BA
Small-glanded:							
D	2	4	3.55±0.27	4.50±0.48	0.85±0.08	2.66±0.21	38.71±2.41
CH	9	16	3.03±0.11	3.71±0.21	1.21±0.05	2.90±0.12	36.90±1.17
MO	3	6	3.49±0.23	4.04±0.39	1.29±0.09	2.91±0.20	37.78±1.98
E	9	15	3.97±0.16	5.12±0.31	0.89±0.04	2.95±0.13	36.94±1.15
V	15	29	3.32±0.09	3.80±0.16	1.14±0.04	2.98±0.09	35.00±0.84
S	10	20	3.07±0.11	4.79±0.25	1.13±0.04	3.03±0.11	36.07±1.01
HO	4	8	2.88±0.15	3.57±0.29	0.91±0.06	3.08±0.18	33.69±1.51
CX	7	14	3.07±0.12	3.99±0.24	1.11±0.05	3.30±0.15	37.98±1.28
BR	2	4	3.46±0.26	4.79±0.54	1.11±0.09	3.31±0.27	37.82±2.40
CO	11	22	3.26±0.10	3.43±0.16	1.15±0.04	3.37±0.12	37.89±0.98
P	7	14	3.11±0.13	3.50±0.21	1.06±0.05	3.37±0.15	35.94±1.18
Tovar*	7	10	3.17±0.15	4.77±0.32	1.06±0.06	3.87±0.20	38.42±1.43
Tovar diallel	412	821	3.22±0.08	5.47±0.13	1.13±0.02	4.15±0.06	37.94±0.60
Large-glanded populations:							
CA	4	6	5.49±0.38	4.20±0.27	1.38±0.09	3.99±0.19	38.50±1.81
C	11	21	5.26±0.19	6.43±0.22	1.35±0.05	4.47±0.12	46.72±1.18
LM	5	9	5.00±0.31	6.07±0.33	1.47±0.08	4.70±0.19	53.94±2.07
Tulum*	9	16	4.66±0.19	5.28±0.20	1.84±0.08	4.70±0.14	41.51±1.22
CP	1	2	5.99±0.68	5.68±0.60	1.91±0.21	4.75±0.39	43.97±3.59
PM	11	22	5.57±0.20	5.36±0.18	1.69±0.06	4.81±0.12	45.06±1.15
GS	12	23	5.15±0.19	5.30±0.17	1.72±0.06	4.90±0.12	42.86±1.03
CC	21	37	6.34±0.19	5.70±0.16	1.32±0.04	5.00±0.10	46.99±0.88
M	10	18	4.86±0.20	5.60±0.21	1.60±0.06	5.07±0.14	47.47±1.22
T	15	25	5.03±0.16	5.67±0.18	1.75±0.06	5.10±0.12	45.40±1.11
CN	2	3	5.93±0.59	6.09±0.59	1.42±0.13	5.51±0.39	51.73±3.39
BA	7	13	5.29±0.24	4.98±0.21	1.71±0.08	6.11±0.20	51.59±1.60
Tulum diallel	562	1042	4.63±0.04	4.61±0.05	1.36±0.01	4.37±0.04	38.19±0.34
Species averages:							
Small glanded			3.24±0.20	4.22±0.43	1.07±0.09	3.27±0.23	36.86±1.04
Large glanded			5.31±0.35	5.64±0.40	1.54±0.12	4.88±0.32	47.60±3.43

* The Tulum and Tovar populations are descendants from the diallel populations, but measured by a different observer. For the species averages these data are used, but not the diallel data.

Table S3. Summary statistics (mean±SE) for the bract traits in the different populations and species (average of the populations). Description of how the parameters were obtained is given in Table S2.

Population	UBLc	UBLs	UBW	LBLc	LBLs	LBW
Small-glanded populations:						
D	19.80±1.23	16.22±0.93	19.86±1.36	21.28±1.46	18.07±1.05	21.57±1.50
CH	18.42±0.57	15.79±0.46	18.57±0.62	20.20±0.70	17.21±0.49	20.27±0.70
MO	18.66±0.99	16.05±0.76	19.39±1.13	20.35±1.18	17.23±0.81	21.27±1.28
E	18.99±0.61	16.20±0.49	17.47±0.61	21.79±0.75	18.55±0.54	19.93±0.72
V	17.86±0.42	15.24±0.33	17.78±0.45	19.15±0.50	16.24±0.36	19.01±0.53
S	17.85±0.50	15.35±0.39	18.55±0.59	19.01±0.59	16.87±0.43	19.77±0.67
HO	17.26±0.74	15.13±0.60	16.97±0.86	18.37±0.87	16.01±0.67	17.81±0.88
CX	19.34±0.66	16.07±0.51	19.46±0.76	20.68±0.76	17.27±0.53	20.91±0.86
BR	17.23±1.09	15.50±0.90	19.87±1.37	20.05±1.40	16.91±0.94	22.02±1.60
CO	18.89±0.49	15.98±0.39	19.90±0.60	20.81±0.60	17.52±0.44	20.61±0.64
P	17.47±0.59	15.33±0.49	19.08±0.72	18.85±0.67	16.31±0.49	19.82±0.75
Tovar*	19.80±0.76	17.28±0.64	19.32±0.85	21.47±0.90	18.36±0.67	19.57±0.91
Tovar diallel	18.97±0.31	16.54±0.26	19.49±0.33	21.24±0.38	18.27±0.32	19.99±0.34
Large-glanded populations:						
CA	19.37±1.07	17.12±0.94	20.03±0.98	20.95±1.11	17.85±0.86	19.95±0.99
C	23.82±0.71	21.31±0.64	23.57±0.61	25.35±0.76	22.02±0.59	24.23±0.69
LM	26.45±1.16	21.67±1.02	27.92±1.12	30.75±1.46	25.60±1.09	30.33±1.29
Tulum*	18.50±0.66	17.22±0.59	22.59±0.66	21.40±0.75	19.06±0.61	22.89±0.73
CP	21.53±1.98	20.32±1.82	23.86±2.05	23.14±2.03	20.57±1.68	21.95±1.94
PM	21.91±0.67	19.98±0.60	23.76±0.60	24.25±0.69	20.67±0.56	23.47±0.61
GS	19.98±0.59	18.33±0.56	23.29±0.61	22.28±0.62	19.26±0.50	23.62±0.64
CC	24.45±0.54	22.55±0.53	23.48±0.48	25.90±0.60	22.66±0.48	23.88±0.53
M	23.89±0.77	22.01±0.72	24.65±0.75	26.31±0.85	22.57±0.65	24.68±0.75
T	20.48±0.58	19.15±0.54	24.11±0.57	24.51±0.67	20.99±0.54	24.81±0.68
CN	25.95±2.07	23.89±1.87	26.71±1.89	27.82±2.14	23.75±1.58	28.10±1.99
BA	25.87±0.97	23.34±0.88	27.62±0.93	26.78±1.02	23.50±0.78	27.19±0.99
Tulum diallel	18.88±0.17	16.63±0.13	20.32±0.18	20.52±0.20	17.38±0.16	20.49±0.22
Species averages:						
Small glanded	18.61±0.58	15.98±0.46	18.78±0.68	20.33±0.80	17.39±0.62	20.01±0.83
Large glanded	23.45±1.92	20.83±1.46	24.76±0.18	26.03±1.92	22.29±1.56	25.40±1.72

* The Tulum and Tovar populations are descendants from the diallel populations, but measured by a different observer. For the species averages these data are used and not the diallel data.

Table S4. Posterior distribution of the Tovar G-matrix for the functional traits, provided in the external file “G_posterior_Tovar_functional_traits.csv” (comma delimited). The traits are on mean standardized scale. Each row is one sample from the joint posterior distribution. The columns are the elements of the G-matrix given row wise (or column vise, the matrix is symmetrical). The format of the column names are best described by an example. The column with name GAD:GAD gives the posterior distribution of the variance in GAD, while the column with name GAD:GSD gives the posterior distribution of the covariance between GAD and GSD. Note that √GA and √BA are simplified to GA and BA. The file can be read into R by the command: `G = read.csv("filename.csv")`. The posterior median of each matrix element can be computed, and printed in matrix format, by the command: `matrix(apply(G, 2, median), ncol = sqrt(ncol(G)))`.

Table S5. Posterior distribution of the Tulum G-matrix for the functional traits, provided in the external file “G_posterior_Tulum_functional_traits.csv”. See table S4 for explanation.

Table S6. Posterior distribution of the Tovar G-matrix for the bract traits, provided in the external file “G_posterior_Tovar_bract_traits.csv”. See table S4 for explanation.

Table S7. Posterior distribution of the Tulum G-matrix for the bract traits, provided in the external file “G_posterior_Tulum_bract_traits.csv”. See table S4 for explanation.

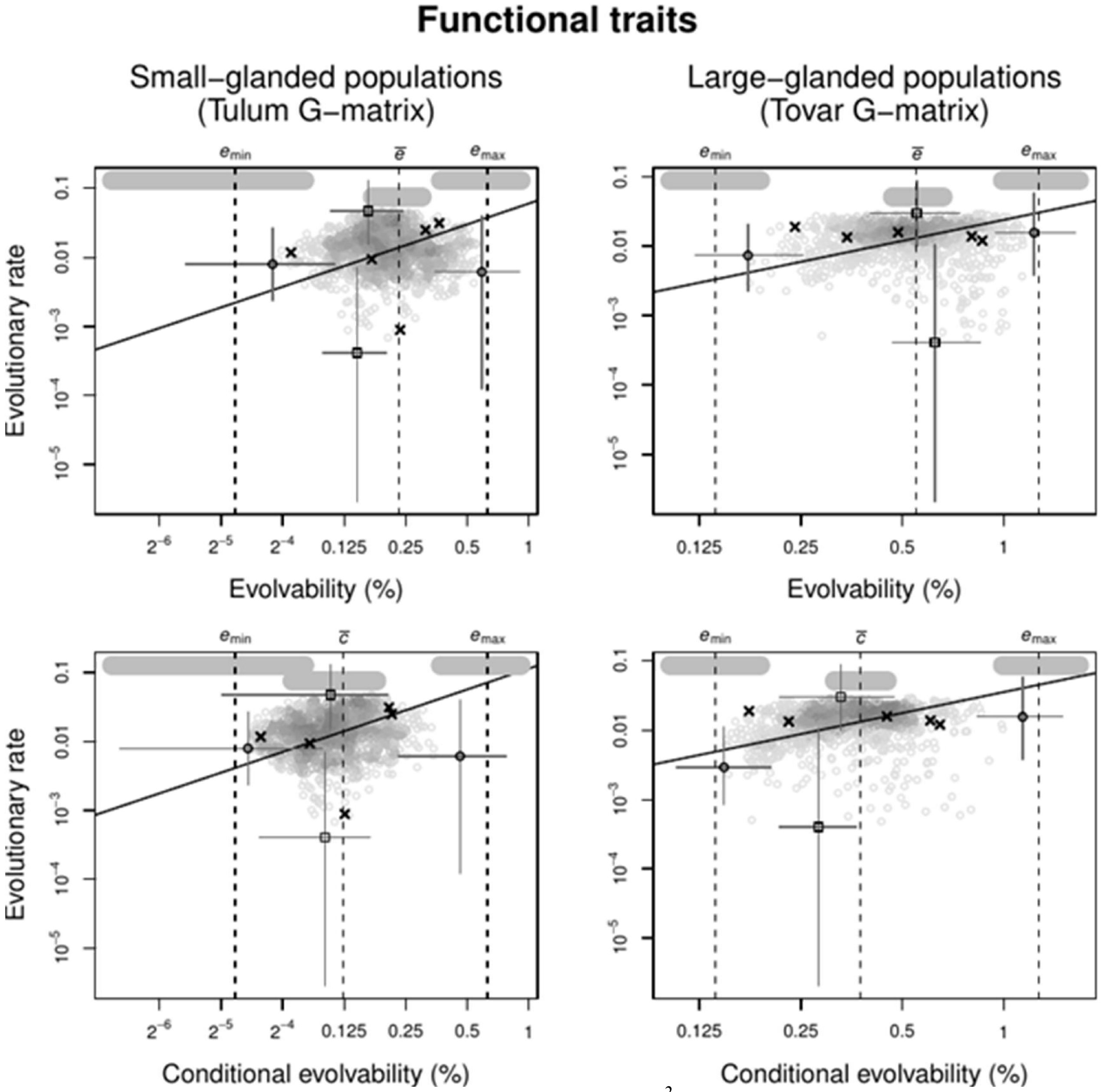

Figure S1 Scaling relationship between evolutionary rate (σ^2_{rate}) and the evolvability estimates for a population of the other species for the functional traits. Grey circles represent 1000 uniformly distributed random directions (selection gradients). The solid line indicates the isometric relationship (a slope of 1) through the mean of the random directions. Crosses are the measured traits, squares are the directions with highest or lowest trait divergence, and black circles are the directions with highest and lowest evolvability out of the 1000 random directions. The circles, crosses, and squares are the modes from the posterior distributions and the grey lines give the 95% highest posterior density intervals. The vertical dotted lines are the posterior modes for the parameters named above each plot, and the vertical thick grey bars are their 95% highest posterior density intervals (see Table 2). The differences between minimum and maximum evolvability and the lowest and highest evolvability of the random directions are partly due to sampling error and partly due to bias in the estimates of $e_{\min}$ and $e_{\max}$.

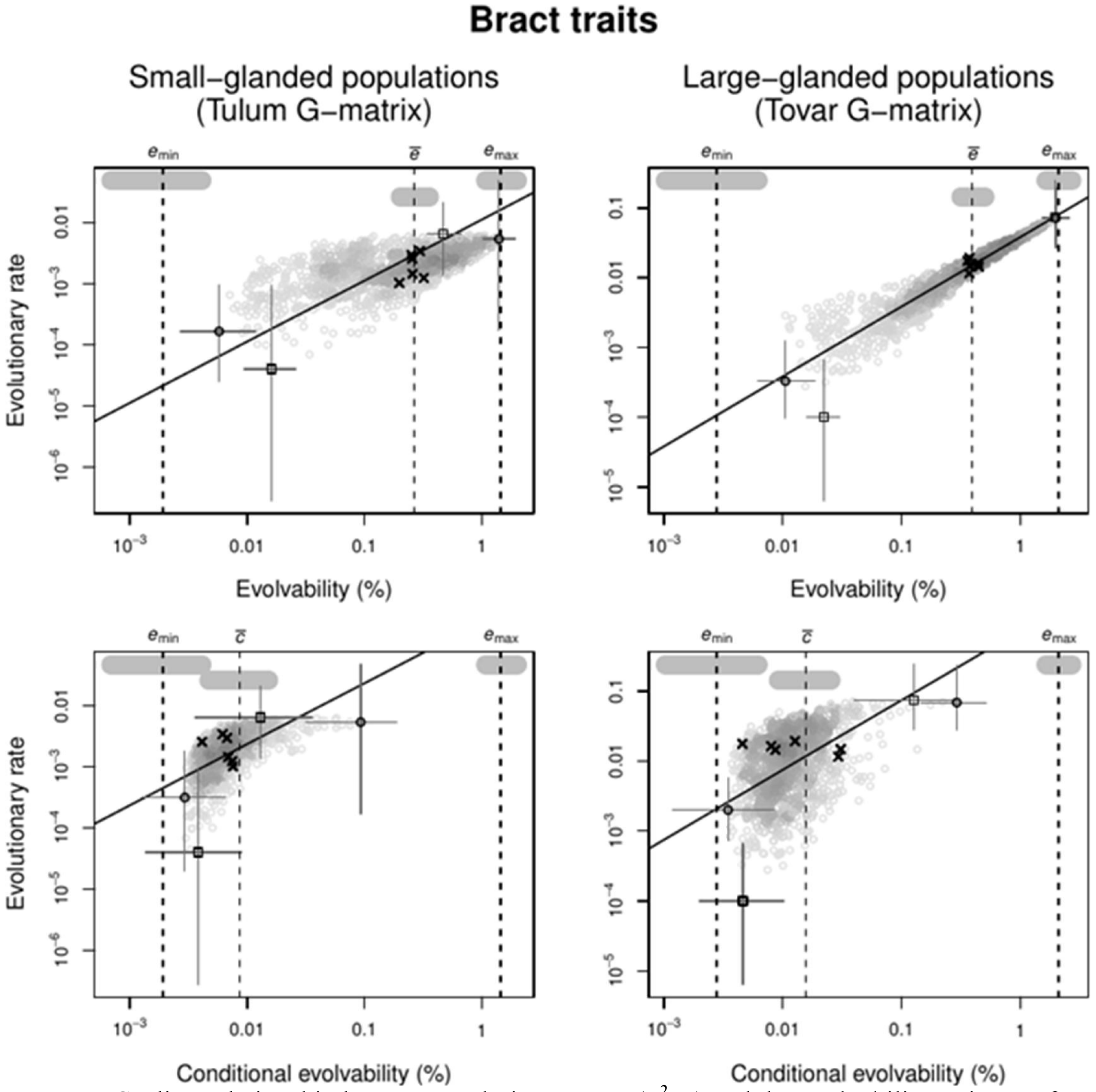

Figure S2 Scaling relationship between evolutionary rate (σ^2_{rate}) and the evolvability estimates for a population of the other species for the bract traits. See Figure S1 for explanation of symbols.

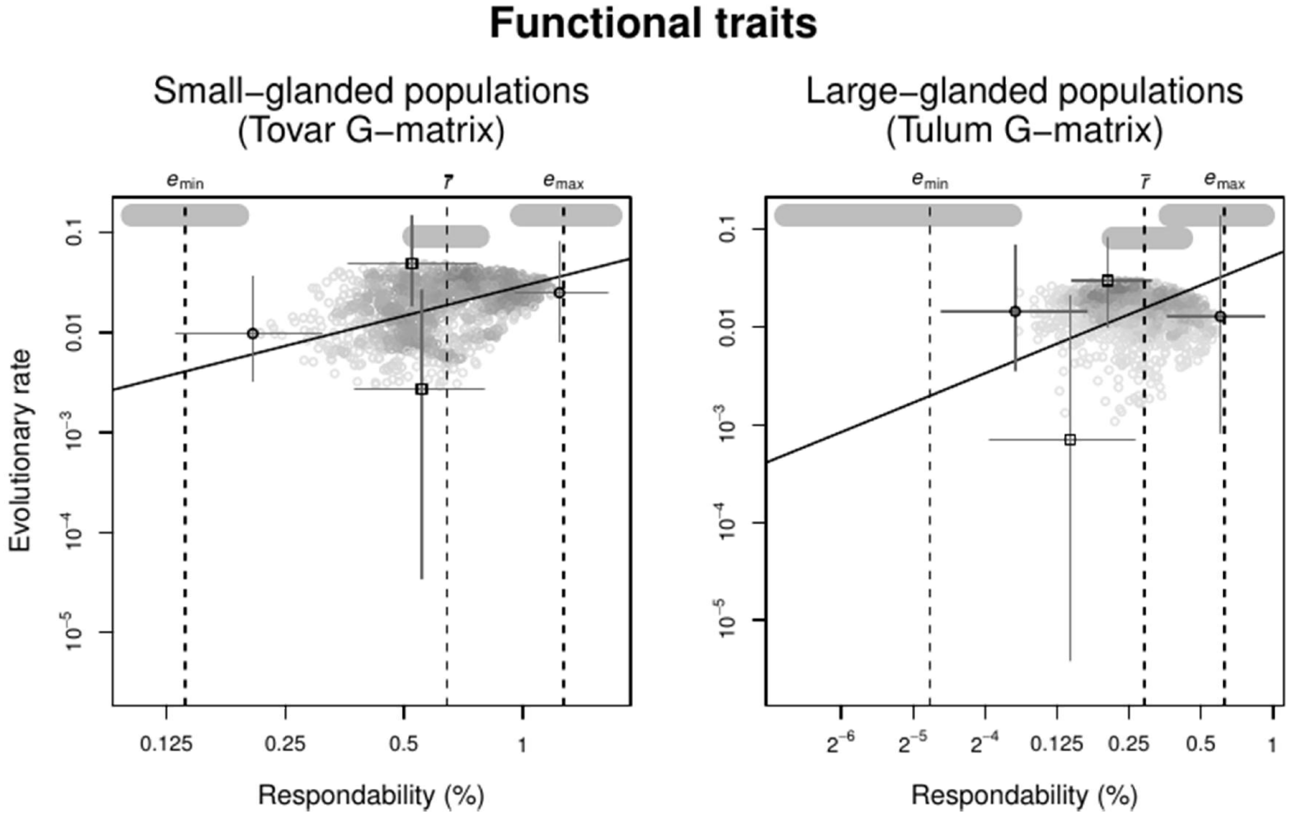

Figure S3. Scaling relationship between respondability, computed along selection gradients, and evolutionary rates (σ^2_{rate}), computed along corresponding response vectors, for the functional traits. Unit length response vectors are given by **Gβ**/||**Gβ**||, where **β** is the column vector of the corresponding unit length selection gradient and ||**x**|| denotes the norm of **x**. See Figure S1 for explanation of symbols. Note that the traits are not included in this figure as we do not have information about the direction of selection.

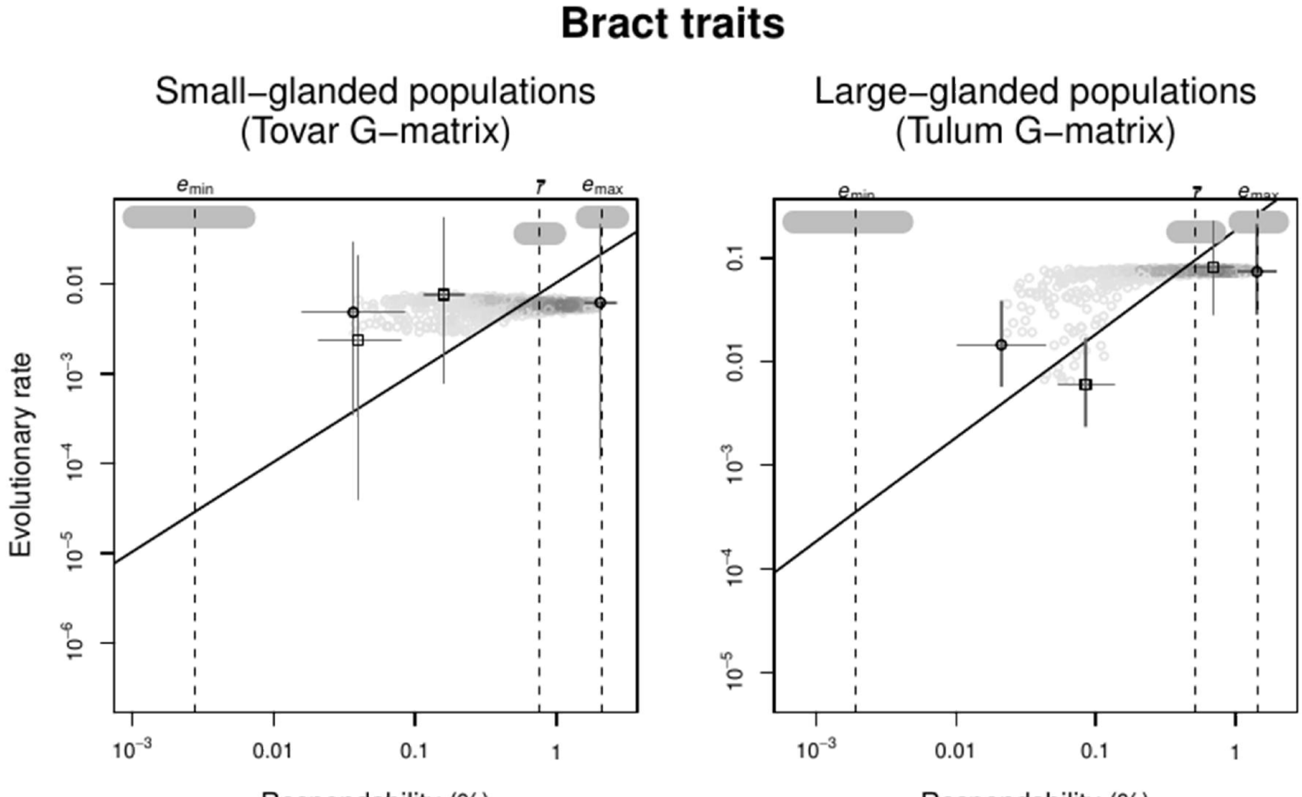

Figure S4. Scaling relationship between respondability, computed along selection gradients, and evolutionary rates (σ^2_{rate}), computed along corresponding response vectors, for the bract traits. See Figure S3 for further explanation.

Appendix A: Tracking optimum model

Here we consider a simplified model of a trait tracking an optimum that moves according to an Ornstein-Uhlenbeck process. We assume the selection gradient on the trait is $\beta = -2s(z-\theta)$, where z is (log) trait mean and θ is the optimum. The parameter s is the (mean-scaled) curvature of an assumed quadratic fitness function. The model is

$$dz = e\beta = -2es(z-\theta)dt,$$
$$d\theta = -\alpha\theta dt + \sigma dB,$$

where dz, $d\theta$, and dt are differentials in the trait mean, optimum and time, respectively, e is the evolvability, dB is white noise (i.e. the stochastic differential of a unit variance Brownian motion), and α and σ are parameters describing the Ornstein-Uhlenbeck process. By use of Ito's formula for stochastic differentiation we can then form the stochastic differentials:

$$dz^2 = 4ez(\theta - z)dt,$$
$$dz\theta = (2es\theta(\theta - z) - \alpha\theta z)dt + \sigma z dB,$$
$$d\theta^2 = (\sigma^2 - 2\alpha\theta^2)dt + 2\sigma\theta dB,$$

By integrating these over the joint distribution of z and θ, we can form differential equations in the first and second moments of the distribution

$$d\mathrm{E}[z] = e\beta = -2es(\mathrm{E}[z] - \mathrm{E}[\theta])dt,$$
$$d\mathrm{E}[\theta] = -\alpha\mathrm{E}[\theta]dt,$$
$$d\mathrm{E}[z^2] = 4es(\mathrm{E}[z\theta] - \mathrm{E}[z^2])dt,$$
$$d\mathrm{E}[z\theta] = (2es\mathrm{E}[\theta^2] - (2es + \alpha)\mathrm{E}[z\theta])dt,$$
$$d\mathrm{E}[\theta^2] = (\sigma^2 - 2\alpha\mathrm{E}[\theta^2])dt,$$

where $\mathrm{E}[x]$ denotes expectation of x. Because the model is a system of linear stochastic differential equations, the distribution of the variables is multivariate normal, and the time development of these moments fully specify the time development of the distribution. These equations can be solved to find the time development of the system, but for the present purpose it suffices with the stationary distribution, which can be found by setting all the differentials to zero and solve for the moments. This gives

$$\mathrm{Var}[z] = V\frac{2es}{2es+\alpha},$$

where $V = \sigma^2/2\alpha$ is the stationary variance of the optimum, θ. Note that if the dynamics of the optimum converges on a white noise, i.e. that $\alpha \to \infty$ and V stays constant, the variance in the trait mean goes to zero. This is because the optimum moves so fast that the trait can not track it. More generally, if the characteristic time of the optimum, $1/\alpha$, is much shorter (i.e. faster) than the characteristic time of the adaptation, $1/2es$, then the variance of the trait mean goes to zero, because the trait mean can not track the movements of the optimum. If, on the other hand, the adaptation is much faster than the movements of the optimum, the trait mean will track the optimum perfectly, and the variance of the trait mean converges on the variance of the optimum, V.

If the Ornstein-Uhlenbeck process for the optimum converges on a Brownian motion, that is, $\alpha \to 0$, and σ is finite, the variance of the optimum is no longer stationary and evolves as $\sigma^2 t$. Under these conditions the variance of the trait mean evolves as

$$\mathrm{Var}[z] = \sigma^2 t - \frac{3\sigma^2}{4es}\left(1 + 3\mathrm{Exp}[-4est] - \frac{4}{3}\mathrm{Exp}[-2est]\right) \approx \sigma^2 t - \frac{3\sigma^2}{4es},$$

where Exp is the exponential function and the approximation is for large *t*. This means that the variance of the trait mean eventually settles on the same rate of increase as the variance of the optimum, but with a constant lag that is inversely proportional to the evolvability.

Appendix B: Construction of the Phylogeny

We constructed the phylogenetic relatedness as follows. Genomic DNA was extracted from leaf tissue using E-Z 96 Pland DNA Kit (Omega Bio-Tek, Norcross, GA, USA). Multiplex PCR reactions were prepared in a total volume of 10 µl containing 5 µl of 2x type-it Microsatellite PCR Kit (QIAGEN, Hilden, Germany), 1 µl of extracted DNA (10 ng) and 1 µl primer (vary in concentration 0.1-0.4 µM). The thermal condition was set to 95 ° C for 5 min for initial denaturation; 10 initial cycles as touchdown at 94 ° C for 30 s, 60–50 ° C for 45 s, 72 ° C for 45 s; 25 cycles at 94 ° C for 30 s, 50 ° C for 45 s, 72 ° C for 45 s; and final extension at 72 ° C for 10 min in an ABI 9600 thermal cycler (Applied Biosystems, Foster City, California, USA). The 5' end of forward primers for 70 microsatellite loci was labeled with four different fluorophores (6-FAM, HEX, NED, and PET); and fragment analysis was conducted with an ABI 3130xl Genetic Analyzer (Applied Biosystems). Microsatellite alleles were scored using GeneMapper software version 4 (Applied Biosystems). Seventy microsatellite markers (Table S8) that have been recently developed in *D. scandens* were screened in 76 individuals (Falahati-Anbaran et al., 2013; Falahati-Anbaran et al., Unpublished). In total 23 Mexican populations from different regions of the country and one Venezuelan population were used in this study (Table S9). A neighbour-joining phylogenetic tree (Saitou & Nei, 1987) was constructed at the population level based on the genetic distance D_A described in Nei et al.(1983) using Populations 1.2.31 program freely available at http://bioinformatics.org/~tryphon/populations/. The branch lengths of the phylogenetic tree were nearly of equal length when the tree was rooted on the centre, and was further ultrametrized by the function "chronopl" in the R-package "ape" (Paradis *et al*. 2004) with the parameter lambda set to unit, which gives a clock-like model.

Table S8 List of microsatellite loci used to construct phylogenetic tree for *Dalechampia scandens* populations.

Locus	Number of alleles	Size range (base pair)
CCdi4	14	190-218
CCdi9	6	102-126
CCdi10	13	199-247
CCdi11	14	304-340
CCdi13	7	98-115
CCdi23	5	159-172
CCdi24	4	133-148
CCdi25	6	220-241
CCdi27	9	68-86
CCdi29	7	53-75
CCdi33	4	180-196
CCdi38	5	106-118
CCdi39-1	20	313-378
CCdi39-2	4	90-96
CCdi41	8	198-231
CCdi45	6	102-127
CCdi47	4	216-236
CCdi50	19	263-316
CCdi52	4	70-78
CCdi53	16	101-133
CCdi54	12	272-301
CCdi63	3	128-132
CCdi67	11	156-181
CCdi71	3	194-200
CCdi74	6	144-173
CCtri1	1	168
CCtri2	6	77-86
CCtri3	4	88-98
CCtri6	13	97-136
CCtri8	3	165-198
CCtri10	4	175-184
CCtri13	11	215-230
CCtri14-1	5	97-119
CCtri14-2	5	78-85
CCtri15	4	124-127
CCtri17	9	207-248
CCtri21	6	138-157

Table S8 Continues.

Locus	Number of alleles	Size range (base pair)
Edi2-1	8	134-147
Edi2-2	13	264-300
Edi5	11	116-139
Edi6	5	179-185
Edi8	2	251-280
Edi9	4	228-246
Edi10	13	239-268
Edi11	2	212-214
Edi13	8	104-126
Edi14	5	138-164
Edi17	19	279-309
Edi20	3	97-101
Edi22	5	96-106
Edi23	9	133-152
Edi33	7	288-321
Edi34	10	86-109
Edi41	11	182-214
Etri1	3	172-177
Etri2	12	264-308
Etri4	1	216
Etri5	4	140-152
Etri9	8	216-237
Etri11	7	262-283
Etri12	5	144-159
Etri13	4	147-157
Etri14-1	3	126-132
Etri14-2	7	183-203
Etri15	6	117-190
Etri16	3	160-166
Etri17	2	88-91
Etet4	1	348
Etet7	4	121-133
Etet12	3	126-134

Table S9 Number of individuals used for microsatellite analyses from *Dalechampia scandens* populations. All populations are distributed throughout Mexico except that Tovar is collected from Venezuela.

Population	Abbreviation	Number of samples
Carillo Puerto	CP	1
Champotón	CN	2
Cacalchén	CA	3
Comalcalco	C	4
Bacalar	BA	4
Graciano Sánches	GS	3
La Mancha	LM	5
Martinez de la Torre	M	4
Tulúm	T	4
Tulúm diallel		2
Puerto_Morelos	PM	4
Ciudad del Carmen	CC	4
Bolonchén de Rejón	BR	1
Hopelchen	HO	2
Motul de Carillo Puerto	MO	3
China	CH	4
Dzemul	D	2
Peto	P	3
Santa Elena	S	3
El Limón	E	4
Chemax	CX	4
Cozumel	CO	4
Valladolid	V	4
Tovar diallel		2

Appendix C: The R-package "evolvability"

The package `evolvability` contains functions for calculating evolvability parameters described in Hansen and Houle (2008, 2009). The package is distributed through http://cran.r-project.org/. Updates and additions of this package will be published on the CRAN package repositories. The following functions are currently implemented:

`evolvabilityMeans` calculates average evolvability, conditional evolvability, respondability, autonomy, integration, maximum evolvability and minimum evolvability from a given G-matrix using the approximation equations in Hansen and Houle (2008)

`evolvabilityMeansMCMC` calculates the same as `evolvabilityMeans`, only over a posterior distribution of a G-matrix.

`evolvabilityBeta` calculates evolvability, conditional evolvability, respondability, autonomy, and integration along each given selection gradient. The function also calculates averages, minimum, and maximum values over a set of given selection gradients.

`evolvabilityBetaMCMC` calculates the same as `evolvabilityBeta`, only over a posterior distribution of a G-matrix.

`evolvabilityBetaMCMC2` calculates evolvability, conditional evolvability, respondability, autonomy, and integration along one selection gradient when both the G-matrix and the selection gradient is given by a posterior distribution.

`randomBeta` generates a set of selection gradients uniformly distributed in a hypersphere of dimension k.

`meanStdG` mean standardizes a G-matrix given the corresponding means. Note that mean standardization is only meaningful for traits on a ratio or log-interval scale. Traits on these scales have a meaningful (unique and non-arbitrary) zero value.

`meanStdGMCMC` mean standardizes the posterior distribution of a G-matrix by the corresponding posterior distribution of the means.

References

Falahati-Anbaran M, Stenøien HK, Pélabon C, Bolstad GH, Perez-Barrales R, Hansen TF, Armbruster WS. 2013. Development of microsatellite markers for the Neotropical vine *Dalechampia scandens* (Euphorbiaceae). *Applications in Plant Sciences* **1**: 1200492.

Falahati-Anbaran M, Stenøien HK, Pélabon C, Bolstad GH, Perez-Barrales R, Hansen TF, Armbruster WS. Unpublished. Novel microsatellite markers for small-glanded populations of the Neotropical vine *Dalechampia scandens* (Euphorbiaceae).

Hansen T, Houle D. 2008. Measuring and comparing evolvability and constraint in multivariate characters. *Journal of Evolutionary Biology* **21**: 1201–1219.

Hansen T, Houle D. 2009. Corrigendum. *Journal of Evolutionary Biology* **22**: 913–915.

Nei M, Tajima F, Tateno Y. 1983. Accuracy of estimated phylogenetic trees from molecular data. *Journal of Molecular Evolution* **19**: 153-170.

Paradis E, Claude J, Strimmer K. 2004. APE: analyses of phylogenetics and evolution in R language. *Bioinformatics* **20**: 289-290.

Saitou N, Nei M. 1987. The neighbor-joining method: a new method for reconstructing phylogenetic trees. *Molecular Biology and Evolution* **4**: 406-425.
